# Night of Two Town Meetings

**DOI:** 10.3201/eid0810.020362

**Published:** 2002-10

**Authors:** Matthew L. Cartter

**Affiliations:** * Connecticut Department of Public Health, Hartford, Connecticut, USA

Driving south through Connecticut’s Naugatuck River Valley brought back memories. My dad used to drive this road to and from work 30 years ago, when the river was actually more polluted but the circumstances were less complicated. On this night, a week after Thanksgiving, the fog seemed inseparable from the road. We were looking for Derby High School, site of our first town meeting. The high school was not far from the hospital where the day before Thanksgiving a 94-year-old woman from nearby Oxford had died of intentionally released inhalational anthrax.

As we pulled into the parking lot, more memories rushed into my mind—I came to this high school with my school’s cross-country team back in 1970 to run a race. The 1970s seem so long ago—smallpox had been eradicated from the world, anthrax was a rare disease called woolsorters’ disease, and the twin towers graced the New York City skyline. In those days, we would be visiting the 94-year-old Oxford woman to study the secrets of her longevity, to see how she had managed to beat life expectancy rates and survive to ripe old age. As our public health team made its way into the building, we were practically run over by a group of students using the hallway as an indoor track. I had to smile—I got my first shin splints running down school halls myself. Besides, it was heartening to see normal school activity in our changed world of post-September 11.

The local health director introduced me as the first of the evening’s speakers to the town officials, legislators, first responders, and a score of townspeople who had braved the foggy evening to attend the meeting. In spite of the bright lights, a different kind of fog hung heavily inside the building. As I approached the podium, I felt the expectations of the people in the audience. They needed specific, practical, relevant information about their predicament as a small community in the middle of a disease outbreak caused by bioterrorism. I had been to many town meetings before, but this meeting was different. In my public health career until now, disease outbreaks had been natural events. Investigators and the community had a defined enemy, a disease. Now, the disease was only part of the problem. Someone had intentionally created this outbreak, and while we knew how to go after viruses and bacteria, we knew little about human perpetrators of disease.

“I am a public health worker involved in the anthrax investigation,” I offered cautiously, “here to discuss the 22 cases of anthrax reported in the United States over the past 2 months, review their possible causes, and propose options and solutions.” My presentation covered the history of the anthrax outbreaks in the District of Columbia, Florida, New Jersey, and New York; the findings of the outbreak investigations; and the findings of the criminal investigations reported in the press. In addition to the usual slides shown by epidemiologists during traditional outbreaks, I showed slides of the letters sent through the postal service to newsman Tom Brokaw and to Senator Daschle. The Brokaw letter had no return address, I elaborated, while the return address on the Daschle letter was a fictitious New Jersey elementary school. I could tell that many in the audience had not looked closely at these handwritten letters before (handwriting is personal and adds to the abomination of these letters).

When my talk came to a close, I explained that those of us from the state health department needed to leave for a town meeting in Oxford. I thanked the audience and the other speakers for their understanding. I heard several people say thanks as I walked up the aisle. I have never had to leave a town meeting early and hoped that things would go well for those talking and answering questions after me.

The fog thickened as we headed out of the valley to a middle school in Oxford. Here, the parking lot was filled with cars and media trucks with satellite dishes, signs of the media frenzy that permeated the anthrax investigation. The meeting was already well under way. I looked through a small window in the door of the gymnasium and took a deep breath before entering—town meetings are unscripted events that can be very unpredictable, especially when the media are present. The public and the media were seated in chairs and bleachers on one side of the room. The speakers, finished with their presentations, were seated at tables on the opposite side. The local health director was standing at the podium responding to questions. As we walked through the door, he seemed relieved to introduce us as “the folks from the state health department.” I sensed that the tension I felt was not all mine.

On my way to the podium, I passed the speakers’ table and nodded to the physician who had treated the elderly anthrax patient. I greeted the First Selectman, who was completing her second week in office. Earlier in the day, she had mentioned to me that some town residents still hoped that this was a case of “natural” anthrax, not connected to recent events. I knew that some in the audience would not be comforted by what I needed to say.

“I am a physician from the state health department,” I began, “and have been part of the public health response to many emergencies: West Nile virus infection, Lyme disease, and now anthrax. I grew up not far from here, in a small town much like Oxford, and my job today is to answer your questions.”

One man wanted to know if we had found anthrax in any of the soil samples we had collected; another how long anthrax from contaminated letters remained viable. A woman asked if it was okay for her children to touch the mail. The concerns and questions were many and far-reaching, but I had settled in and did not feel a need to hurry. The press seemed anxious to meet their deadlines, but this meeting was not for them. Earlier in the afternoon, a report had been released on the status of the anthrax investigation. I gave a brief analysis of the report. The source of exposure to *Bacillus anthracis* for the elderly Connecticut resident remains unknown, the report said. The genetic characteristics of *B. anthracis* isolated from the patient were similar to those found in other bioterrorism-related cases; however, the epidemiologic characteristics and the potential sources of exposure were different (1).

“Although we will probably never know exactly how your elderly neighbor became infected,” I explained, “she was probably exposed to mail contaminated with anthrax spores. The mail threat, at least this episode, will pass with time, but those of you who live in Oxford, where she lived, may never feel the same about opening the mail.”

As the questions subsided and the meeting came to a close, I made my way through the circle of officials and the dwindling crowd to the door, where the reporters awaited with questions about the newly released report. Finally outside the building, I felt the cool evening air with relief. Even the fog seemed less ominous. “How did it go?” asked one of my colleagues from the state health department as we walked away from the school. “These folks have been through a lot,” I said, “and I feel privileged to be here.”

I left the world of clinical medicine 18 years ago and went to work at the state health department in Connecticut, at first, as part of Centers for Disease Control and Prevention’s Epidemic Intelligence Service. A few years later, a friend gave me an article written by a physician about what it can be like to work in public health (2). I have given a copy of this article (A Piece of My Mind. Have You Ever Practiced Medicine?) to all medical epidemiologists I have supervised.

On the way home from Derby and Oxford, I felt proud to be part of the public health response to bioterrorism. I thought about how physicians in public health still struggle, on occasion, with the question, “Have you ever practiced medicine?” On the night of two town meetings, I knew that the answer was “yes” to the question, “Have you ever practiced public health?”

## References

[1] Centers for Disease Control and Prevention. Update: investigation of bioterrorism-related inhalational anthrax—Connecticut, 2001. MMWR. 2001;50:1049–51. 11808925

[2] Dandoy S. A piece of my mind. Have you ever practiced medicine? JAMA. 1988;260:2113. 10.1001/jama.260.14.2113 3418879

